# Breast lesions classified as probably benign (BI-RADS 3) on magnetic resonance imaging: a systematic review and meta-analysis

**DOI:** 10.1007/s00330-017-5127-y

**Published:** 2017-11-22

**Authors:** Claudio Spick, Hubert Bickel, Stephan H. Polanec, Pascal A. Baltzer

**Affiliations:** Department of Biomedical Imaging and Image-guided Therapy, Medical University of Vienna, Waehringer-Guertel 18-20, 1090 Vienna, Austria

**Keywords:** Breast, Magnetic resonance imaging, Meta-analysis, Diagnostic imaging, BI-RADS classification

## Abstract

**Purpose:**

To investigate prevalence, malignancy rates, imaging features, and follow-up intervals for probably benign (BI-RADS 3) lesions on breast magnetic resonance imaging (MRI).

**Methods:**

A systematic database-review of articles published through 22/06/2016 was performed. Eligible studies reported BI-RADS 3 lesions on breast MRI. Two independent reviewers performed a literature review and data extraction. Data collection included study characteristics, number/type of BI-RADS 3 lesions, final diagnosis (histopathology and/or follow-up). Sources of bias (QUADAS-2) were assessed. Meta-analysis included data-pooling, heterogeneity testing, and meta-regression.

**Results:**

Fifteen studies were included. Prevalence was reported in 11 studies (range: 1.2-24.3%). Malignancy rates ranged between 0.5-10.1% (pooled 61/2814, 1.6%, 95%-CI:0.9-2.3% (random-effects-model), I^2^=53%, P=0.007). In a subgroup of 11 studies (2183 lesions), highest malignancy rates were observed in non-mass lesions (pooled 25/714, 2.3%, 95%-CI:0.8-3.9%, I^2^=52%, P=0.021) followed by mass lesions (pooled 15/771, 1.5%, 95%-CI:0.7-2.4%, I^2^=0%, P=0.929), and foci (pooled 10/698, 1%, 95%-CI:0.3-1.7%, I^2^=0%, P=0.800). There was non-significant negative association between prevalence and malignancy rates (P=0.077). Malignant lesions were diagnosed at all follow-up time points.

**Conclusion:**

While prevalence of MRI BI-RADS 3 lesions was strongly heterogeneous, pooled malignancy rates met BI-RADS benchmarks (<2%). Malignancy rates varied, exceeding 2% in non-mass lesions. Twenty-four-month surveillance is required to detect all malignant lesions.

***Key points*:**

*• Probably benign (BI-RADS 3) lesions showed a pooled malignancy-rate of 1.6% (95%-CI:0.9-2.3%).*

*• Malignancy rates differ and are highest in non-mass lesions (2.3%, 95%-CI:0.8-3.9%).*

*• The prevalence of BI-RADS 3 lesions on breast MRI ranged from 1.2-24.3%.*

*• Malignant lesions were diagnosed at follow-up time points up to 24 months.*

## Introduction

The Breast Imaging and Reporting Data System (BI-RADS) has been published by the American College of Radiology in order to provide a standardised description and categorisation of breast lesions on mammography, ultrasound, and magnetic resonance (MR) imaging [1].

Breast lesions classified as probably benign (BI-RADS 3) on MR imaging should have a less than two percent frequency of malignancy. These lesions should undergo short-term follow-up with an appropriate methodology to exclude malignancy, rather than being biopsied. The probably benign (BI-RADS 3) category in breast MR imaging is assigned based on the reporting radiologist’s discretion. Consequently, the resulting malignancy rates and specific imaging features of probably benign (BI-RADS 3) breast lesions on MR imaging remain a matter of debate [1].

Several studies have evaluated probably benign lesions on breast MR imaging. Investigated BI-RADS 3 characteristics included prevalence, malignancy rates, and imaging features. These studies diverged with regard to technical aspects, study populations, and reader experience. In addition, indications for MR imaging in these studies differed, ranging from high-risk screening, to problem-solving, and breast cancer staging [2, 3]. For an evidence-based approach to patient management in probably benign (BI-RADS 3) lesions on MR imaging, a systematic review and meta-analysis is warranted.

The purpose of this study was to investigate prevalence, malignancy rate, imaging features, and follow-up intervals of breast lesions assigned as probably benign (BI-RADS 3) on breast MR imaging.

## Materials and Methods

This meta-analysis adheres to the Preferred Reporting Items for Systematic Reviews and Meta-Analyses [4]. The protocol for this systematic review and meta-analysis has been prospectively registered with the PROSPERO International register of systematic reviews (registration number CRD42014013441).

### Search strategy

A computerised search was performed using the Pubmed and Scopus database, including all articles listed till 22/06/2016, as no lower time-point limit was defined. The following search terms were used: “probably benign;” “BIRADS 3;” “BI-RADS 3;” “breast magnetic resonance imaging;” “breast MRI.” No language restrictions were applied. The titles and abstracts of search results were reviewed and the full text of eligible studies was retrieved. Since no specific Medical Subject Headings (MeSH) terms for this systematic literature study were identified, additional results were obtained by backward snowballing [5].

All literature searches, study selection, and data extraction were performed by two independent reviewers (CS, six years of experience in breast imaging; PAB, fourteen years of experience in breast imaging). Results after every search and analysis step were compared and discrepancies were solved in consensus. If no consensus was reached, a third reader (HB, six years of experience in breast imaging) served as an arbitrator.

### Eligibility criteria for study selection

Eligibility (inclusion) criteria for study selection were as follows: peer-reviewed studies on female patients undergoing breast MR imaging in whom there were reported probably benign (BI-RADS 3) breast lesions identified by breast MR imaging. A reference standard had to be established either by histopathologic sampling or imaging follow-up of at least 12 months. Not eligible (excluded) were studies on less than 10 subjects, or review articles or studies that had investigated the use of breast MR imaging for other reasons (e.g., probably benign mammographic lesions). A study was included if data about the number and final diagnosis of BI-RADS 3 lesions assessed by magnetic resonance imaging could be extracted. No further restrictions were used.

### Data collection and quality assessment

Data collection included the following parameters: publication year; study design (retrospective vs. prospective); number of observers reading MR imaging; number of patients; age; inclusion criteria for breast MR imaging; number of benign and malignant lesions; lesion size on MR imaging; lesion type (imaging features); MR imaging system type; applied field strength.

One reader applied Quality Assessment of Diagnostic Accuracy Studies (QUADAS-2) items to assess study quality and likelihood of bias. The second reader controlled the results [6]. If present, disagreement was solved in consensus.

### Statistical analysis

All analyses were performed using the software programs OpenMetaAnalyst [7] and STATA 14 (Statacorp, USA). Data pooling was performed using binary random effects models with the DerSimonian-Laird method. Between-study heterogeneity was tested by I^2^-statistics and interpreted as low (≤25%), medium (≤50%), or high (≤75%) [8]. Subgroup analyses were performed using publication year and lesion features on MR imaging (mass, non-mass, or focus) as splitting variables. Meta-regression using a random effects model was used to test for an association between the prevalence of BI-RADS 3 lesions and the respective malignancy rate of these lesions. Publication bias was assessed by Egger´s Funnel plot analysis using metaprop and confunnel commands in STATA. The metatrim command was used to calculate bias-corrected estimates according to the trim and fill method [9]. P-values ≤0.05 were considered significant. P-values were not adjusted for multiple comparisons.

## Results

### Study characteristics and risk of bias assessment

**Fig. 1:**
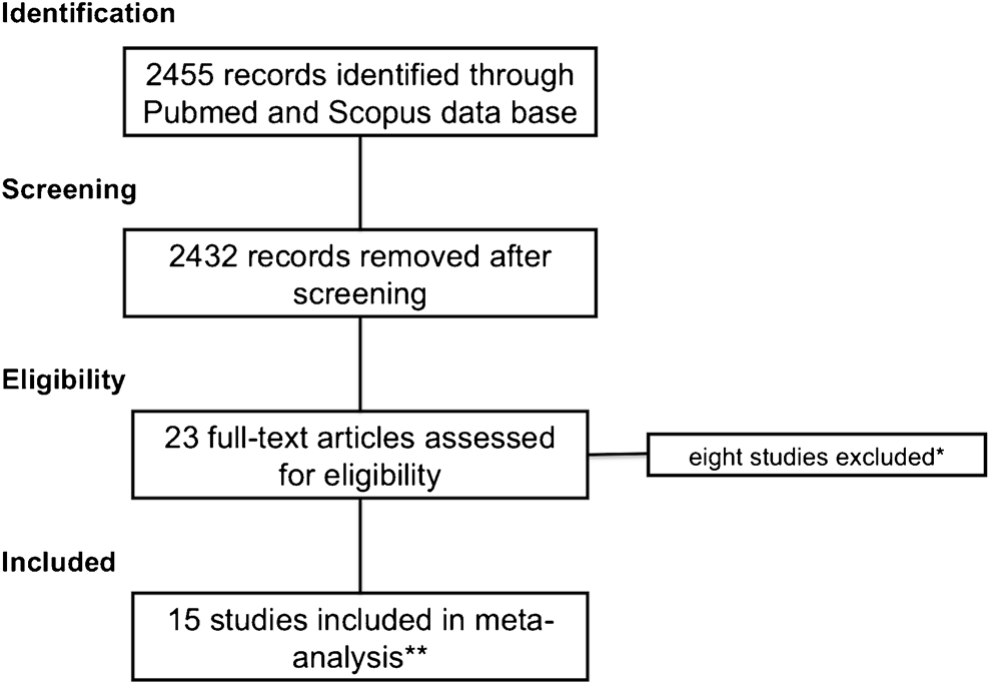
Flowchart shows details on the selection process for the 15 studies ultimately included in the meta-analysis. *: excluded due to insufficient data or review manuscripts without original data. **: [10–24]

**Fig. 2:**
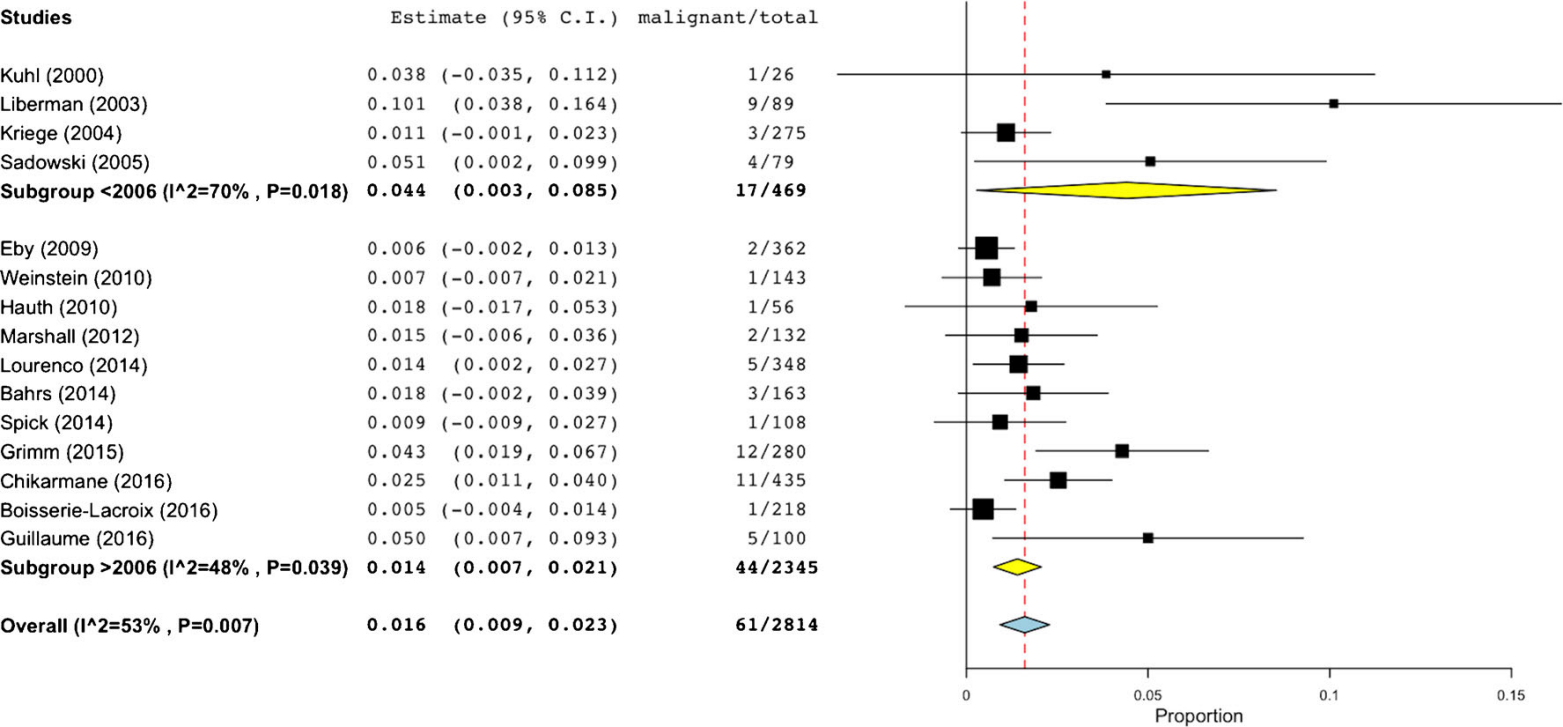
Forest plot of malignancy rates of all studies included in the meta-analysis. Subgroup analysis demonstrates that the majority of outliers in terms of malignancy rates exceeded the BI-RADS benchmark of 2%, having been published before 2006

**Fig. 3:**
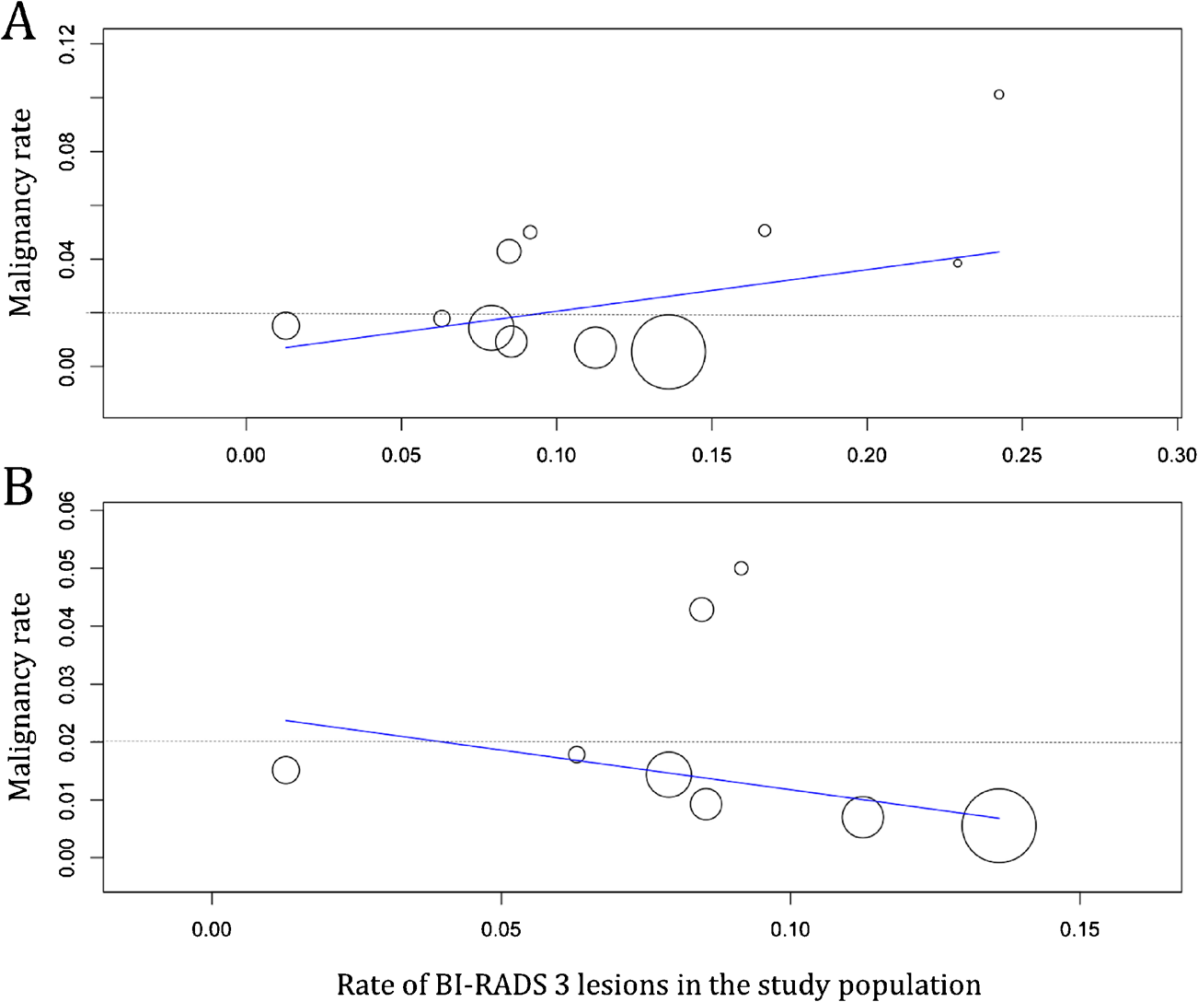
Meta-regression results on the influence of BI-RADS 3 prevalence on BI-RADS 3 malignancy rates (A). Removing outlier studies published before 2006 (B, see also figure 2) revealed a non-significant trend (P=0.077) of lower malignancy rates in studies that reported a higher prevalence of BI-RADS 3 lesions in their study populations. Circle diameters represent study weights according to the random effects model

Overall, 15 eligible studies were selected (Figure 1, Figure 2, Table 1, Table 2) [10–24]. In total, 2814 lesions were included in our meta-analysis (61 showing a malignant outcome). The prevalence of BI-RADS 3 lesions was reported in 11 studies, and ranged from 1.2-24.3% (Figure 3).

**Table 1:** Study characteristics as reported by the publications included in the meta-analysis

First author, year	QUADAS 2	QUADAS 2 Applicability concerns	Patients	BI-RADS 3 Patients	Age (range)	Number of lesions	Number of malignant lesions)	MR imaging indication	MR imaging vendor	MR imaging (Tesla)
	Risk of bias									
Kuhl, 2000 [10]	no	no	192	44	38 (18-65)	26	1	High-Risk Screening	Philips	1.5T
Liberman, 2003 [11]	no	no	367	89	50 (23-82)	89	9	High-Risk Screening	GE	1.5T
Kriege, 2004 [12]	I*/R*	no	1909	N/A	40 (19-72)	275	3	High-Risk Screening	n.a.	N/A
Sadowski, 2005 [13]	no	no	473	79	N/A (26-80)	79	4	Problem-solving	GE	1.5T
Eby, 2009 [14]	no	no	1735	236	50 (28-80)	362	2	Mixed	GE	1.5T
Weinstein, 2010 [15]	no	no	969	109	50 (20-85)	143	1	Screening contralateral breast	N/A	1.5T and greater
Hauth, 2010 [16]	no	no	698	44	50 (21-84)	56	1	Mixed, no high-risk	Siemens	1.5T
Marshall, 2012 [17]	no	no	10342	132	50 (25-81)	132	2	Mixed	GE and Siemens	1.5T
Lourenco, 2014 [18]	no	no	4370	345	49 (25-84)	348	5	Mixed, mainly high-risk	GE and Siemens	1.5T
Bahrs, 2014 [19]	no	no	N/A	117	51 (33-76)	163	3	Mixed	Philips	1.5T
Spick, 2014 [20]	no	no	1265	108	50 (18-89)	108	1	Mixed, no high-risk	Siemens	1.5T
Grimm, 2015 [21]	no	no	3131	265	50 (N/A)	280	12	Mixed	GE and Siemens	1.5T + 3T
Chikarmane, 2016 [22]	no	no	435	435	47.2 (18-85)	435	11	Mixed	GE and Siemens	1.5T + 3T
Boisserie-Lacroix, 2016 [23]	no	no	N/A	101	N/A (23-77)	218	1	Mixed	Philips and Siemens	1.5T
Guillaume, 2016 [24]	I*	no	820	75	43 (26-74)	100	5	Mixed, mainly high-risk	Philips and GE	1.5T + 3T
Total						2814	61			

*: denotes unclear risk of bias, I: index test, R: reference standard, N/A: information not available, GE: General Electric

**Table 2:** Malignant BI-RADS 3 lesions stratified by time of diagnosis

First Author, Year	Diagnosis	Immediate	≤6 months	≤12 months	≤24 months	>24 months	Total
Kuhl, 2000 [10]	invasive	0	1	0	0	0	1
	non-invasive						0
Liberman, 2003 [11]	invasive	0	N/A	N/A	4	0	4
	non-invasive	2	N/A	N/A	3	0	5
Kriege, 2004 [12]*	invasive	N/A	N/A	N/A	N/A	N/A	N/A
	non-invasive	N/A	N/A	N/A	N/A	N/A	N/A
Sadowski, 2005 [13]	invasive	0	0	0	4	0	4
	non-invasive						0
Eby, 2009 [14]	invasive						0
	non-invasive	1	N/A	N/A	1	0	2
Weinstein, 2010 [15]	invasive						0
	non-invasive	1	N/A	N/A	N/A		1
Hauth, 2010 [16]	invasive	0	1**	0	0	0	1**
	non-invasive						0
Marshall, 2012 [17]	invasive	0	0	0	0	1	1
	non-invasive	0	0	1	0	0	1
Lourenco, 2014 [18]	invasive	1	1	1	1	0	4
	non-invasive	1	0	0	0	0	1
Bahrs, 2014 [19]	invasive	0	0	1	0	0	1
	non-invasive	0	0	2	0	0	2
Spick, 2014 [20]	invasive	0	0	0	1	0	1
	non-invasive						0
Grimm, 2015 [21]	invasive	4	0	2	2	1	9
	non-invasive	1	0	0	1	1	3
Chikarmane, 2016 [22]	invasive	0	5	2	1	0	8
	non-invasive	0	2	0	1	0	3
Boisserie-Lacroix, 2016 [23]	invasive	0	0	1	0	0	1
	non-invasive						0
Guillaume, 2016 [24]	invasive	1	2	1	0	0	4
	non-invasive	0	1	0	0	0	1

*: three malignant lesions without information on type or time of diagnosis; **: one phylloid malignant lesion

No researchers in the eligible studies applied predefined criteria for BI-RADS 3 lesions, such as a combination of specific morphological features, kinetic enhancement criteria, and clinical information (e.g., a newly diagnosed lesion), but followed the empirical guidance provided by the BI-RADS lexicon.

**Fig. 4:**
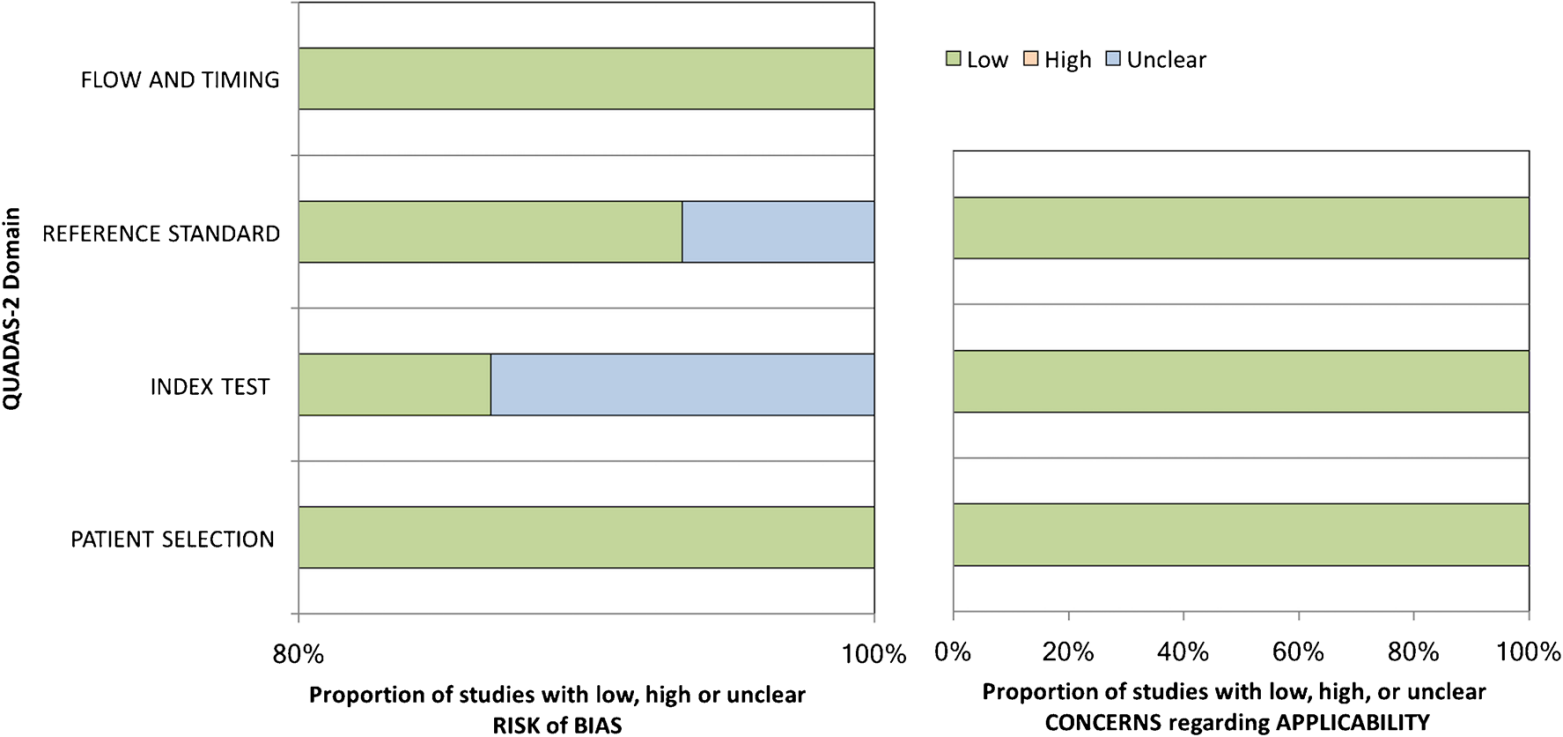
QUADAS-2 graph demonstrates the risk of bias and the applicability of assessment results

The study designs were described as prospective in four [10, 12, 15, 21] and retrospective in 11 studies [11, 13, 14, 16–20, 22–24]. Patient recruitment was consecutive in all included studies. Histopathology and or follow-up was used as a reference standard in all studies. Except for one study [12], all eligible studies provided technical information on MR imaging (Table 1). However, this information was incomplete (e.g., missing spatial and temporal resolution, contrast medium, injection procedure, type of breast coil) in the majority of the studies, and four studies [15, 21, 22, 24] investigated their patients on several devices with varying protocols and field strengths (Table 1). The number of readers was not provided in three articles [12, 13, 24]. Reader experience in breast imaging was given in only six studies [11, 13, 18, 21–23], while four [16, 17, 19, 20] studies specifically reported breast MR imaging experience. The remaining studies did not report, or report insufficient details, about reader experience [10, 12, 14, 15, 24]. Inter-observer variability based on kappa analysis was not assessed in any study. Four studies were performed before 2006 [10–13] and the remaining 11 studies after 2006 [14–24]. Twelve out of 15 studies had a follow-up of 24 months or more [12–16, 18–24] while three had a shorter follow-up [10, 11, 17]. The QUADAS-2 assessment (Figure 4) revealed a low risk of bias in most examined studies. One study was assigned an unclear risk of bias with regard to the reference standard assessment due to a lack of details [12], and two studies were assigned an unclear risk of bias with regard to the conduct of the index test and test interpretation due to a lack of details [12, 24]. No concerns about the ability of the eligible studies to answer the research questions were raised (Figure 4).

### Malignancy rates in BI-RADS 3 lesions

**Fig. 5:**
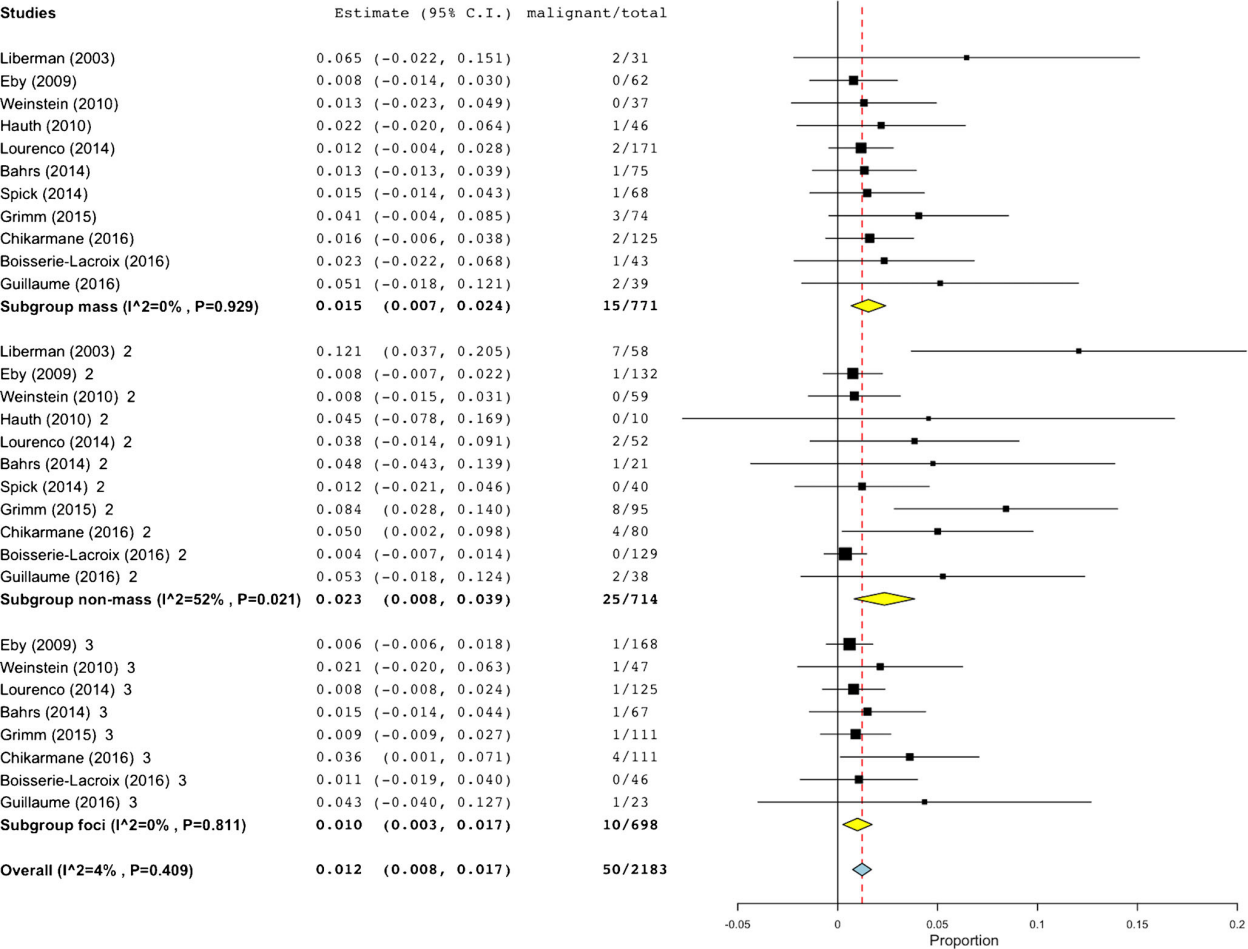
Forest plot of malignancy rates from 11 studies reporting BI-RADS 3 lesion features (mass, non-mass, focus), including subgroup analysis

**Fig. 6:**
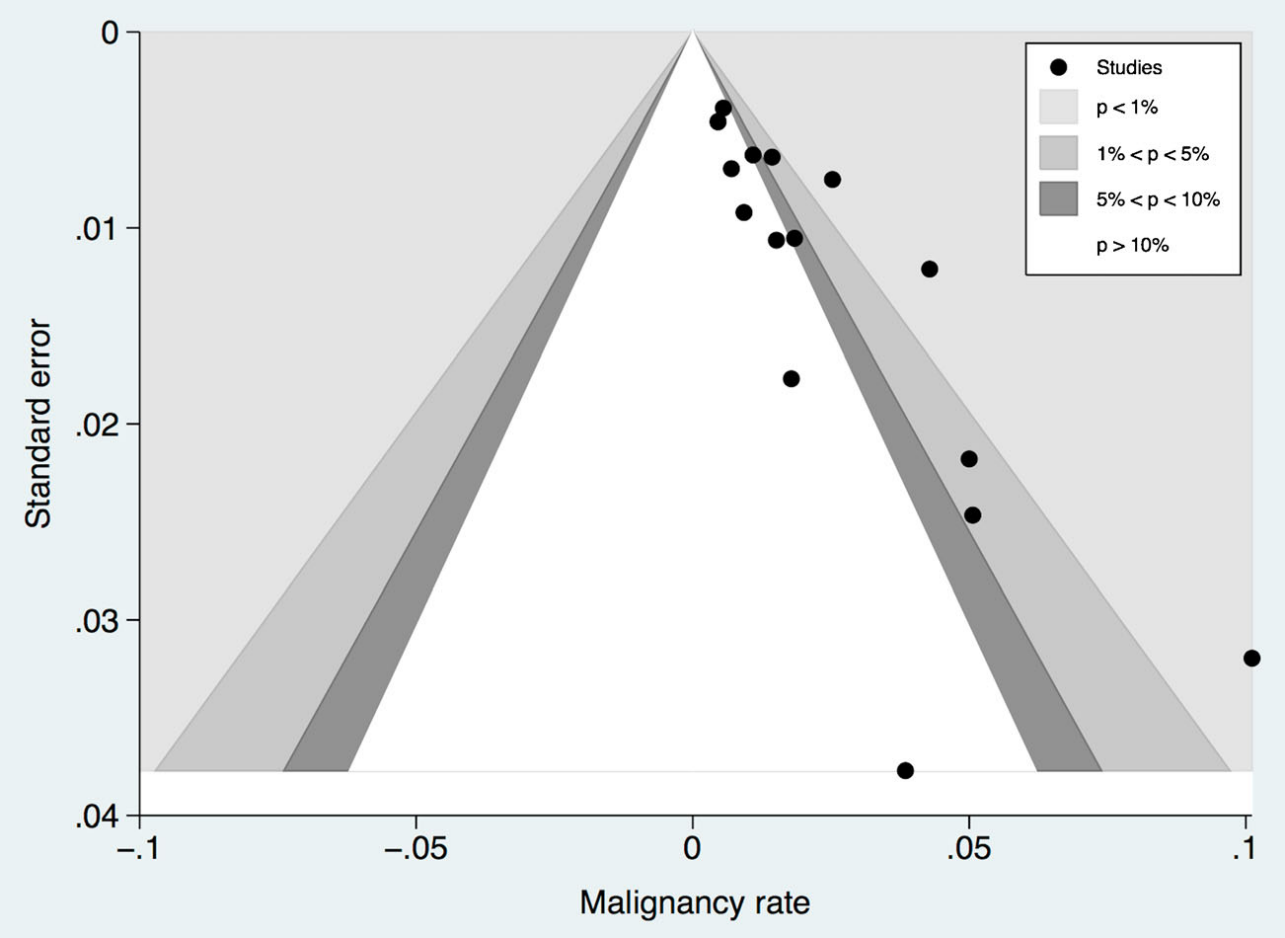
Funnel plot of malignancy rate estimates plotted against their respective standard errors. Funnel contours reflect P-value levels. A publication bias towards higher malignancy rates that is independent from study size is evident as reflected by study points exceeding the funnel contours in the right part of the diagram. Egger´s test confirmed this bias (P<0.001)

The general rate of malignancy was found to be highly heterogeneous (I2=53%, P=0.007, Figure 2) and ranged between 0.5 – 10.1% (61/2814, pooled by random effects model: 1.6%, 95%-CI: 0.9-2.3%). Studies published before 2006 showed a higher rate of malignancy (17/469, pooled by random effects model: 4.4%, 95%-CI: 0.3-8.5%, I2=70%, P=0.018, Figure 2). Studies published after 2006 showed 44/2345 malignant lesions, resulting in a pooled (random effects model) malignancy rate of 1.4%, 95%-CI: 0.7-2.1%, I2=48%, P=0.039, Figure 2. A subset of 11 studies that included 2183 lesions reported on BI-RADS 3 lesion features on MR imaging (malignancy rate 50/2183, Figure 5): the highest malignancy rate was observed in non-mass lesions (25/714, pooled by random effects model: 2.3%, 95%-CI: 0.8-3.9%, I^2^=52%, P=0.021), followed by mass lesions (15/771, pooled by random effects model: 1.5%, 95%-CI 0.7-2.4%, I^2^=0%, P=0.929), and foci (10/698, pooled by random effects model: 1%, 95%-CI 0.3-1.7%, I^2^=0%, P=0.800). Further lesion features, such as morphology and kinetic enhancement characteristics, were provided by only two studies [20, 21], thus precluding further systematic conclusions. There was a non-significant negative association between prevalence and malignancy rates in studies published after 2006 (P=0.077, Figure 3). Egger´s test revealed a significant publication bias (P<0.001) towards higher malignancy rates that was independent from study size (Figure 6). The bias-corrected estimate for the malignancy rate in BI-RADS 3 lesions considering 7 pseudo studies calculated by the trim and fill method (21 degrees of freedom) was 0.9%, 95%-CI: 0.1-1.7%.

### Malignant diagnosis in BI-RADS 3 lesions stratified by time of diagnosis

Of the 61 malignant lesions diagnosed within the included studies, the time of diagnosis was documented in 58 of these, as one study did not provide any details ([12], Table 2). In the investigated studies, there was insufficient data given on whether six-month intervals were strictly applied and how many patients adhered to the follow-up recommendations. Of the 58 lesions, 12 (20.7%, six invasive, six non-invasive cancers) were diagnosed during the immediate work-up, such as second-look (targeted) ultrasound. Another 13 (22.4%, 10 invasive, three non-invasive cancers) were diagnosed after six months and a further 11 lesions (19.0%, eight invasive, three benign) were diagnosed after 12 months’ follow-up. Finally, 19 (32.8%, 13 invasive and six non-invasive cancers) were diagnosed within 24 months after the initial breast MR imaging, and another three lesions (5.2% 2 invasive, one non-invasive cancers) more than 24 months after the initial MR imaging scan (Table 2).

## Discussion

The current systematic review and meta-analysis investigated the prevalence, malignancy rates, imaging features, and follow-up intervals of 2814 breast lesions assigned as probably benign (BI-RADS 3) on breast MR imaging. While the prevalence of MR imaging BI-RADS 3 lesions was highly variable, pooled malignancy rates for BI-RADS 3 lesions on MR imaging met BI-RADS benchmarks (<2%). Malignant lesions were diagnosed at all follow-up time points, currently underscoring the need for 24-month follow-up examinations.

### Prevalence of MR imaging BI-RADS 3 lesions and malignancy rates of MR imaging BI-RADS 3 lesions

While the general pooled malignancy rates of BI-RADS 3 lesions on MR imaging met BI-RADS benchmarks (<2%), the rate of BI-RADS 3 compared to all examined lesions was found to be highly heterogeneous, ranging from 1.2-24.3% of all examined lesions. We explored both the heterogeneity of BI-RADS 3 prevalence and BI-RADS 3 malignancy rates and found that three of four studies published before 2006 exceeded BI-RADS benchmarks regarding malignancy rates. A minor but significant publication bias towards higher malignancy rates independent from study size was identified, as three of these studies published before 2006. Moreover, a lower threshold for calling a benign lesion BI-RADS 3 rather than BI-RADS 2, as reflected by a higher BI-RADS 3 rate, seems to be associated with a lower malignancy rate, as demonstrated in our meta-regression analysis after excluding the aforementioned outliers. These outliers may be due to data acquisition before or shortly after the first MRI BI-RADS edition or older imaging equipment. It needs to be mentioned that the three studies reporting a follow-up period of less than 2 years infer a potential bias towards higher malignancy rates. Considering the overall results reported here, this bias is rather negligible. Of note, two of these studies reported malignancy rates above the average [10, 11].

### Imaging features

Malignancy rates varied according to lesion features, slightly exceeding 2% in non-mass lesions, while being below 2% in both mass and foci. One of the underlying studies revealed that eight (8.4%, 8/95) BI-RADS 3 non-mass lesions were malignant. All of these non-mass lesions presented with heterogeneous or clumped internal enhancement or showed wash-out kinetics [21]. Thus, BI-RADS 3 should be assigned only to non-mass lesions that show nonspecific benign features (e.g., focal/regional homogeneous or slightly heterogeneous enhancement) on baseline MR imaging. The same holds true for masses: lesions with suspicious features, particularly if they present with non-circumscribed or spiculated margins, are associated with a substantial risk of being malignant [25–27] and should not be classified as probably benign (BI-RADS 3).

We did not find a definite set of features that define MR imaging BI-RADS 3 lesions in the evaluated literature. Care should be taken when transferring conventional mammography and ultrasound criteria directly to breast MR imaging: despite the absence of malignant criteria, a newly diagnosed benign lesion regularly requires follow-up to exclude cancer. MR imaging however, offers functional information on tissue composition and vasculature and should thus allow the reader to assign MR imaging BI-RADS 2 in a subset of patients. Cancers that lack all three MR imaging hallmarks of malignancy (non-circumscribed or spiculated margins, plateau or wash-out curve types, and restricted diffusivity) have not been described in the current breast MR imaging literature. Thus, BI-RADS 2 may be assigned to circumscribed lesions that present with persistent enhancement [28–30]. In addition, several authors have documented that high diffusivity as measured by diffusion-weighted imaging (DWI) may reliably exclude breast cancer [31–35]. Finally, non-enhancing lesions are associated with a very low prevalence of malignancy and may thus be classified BI-RADS 2 [30]. Consequently, and by definition, MR imaging BI-RADS 3 should be assigned to lesions that fall into neither BI-RADS 2 (definitely benign) nor BI-RADS 4 (suspicious) categories. Still, a set of imaging features that defines an MR imaging BI-RADS 3 lesion needs to be defined.

### Clinical management and follow-up intervals in MR IMAGING BI-RADS 3 lesions

The results obtained in this systematic review and meta-analysis reveal some suggestions for the clinical management of MR imaging BI-RADS 3 lesions; a substantial rate, 12 of 58 (20.7%) malignant lesions that could be analysed in this respect, were diagnosed during the immediate work-up, including second-look ultrasound. These MR-directed ultrasound upgrades of MR imaging BI-RADS 3 [18, 21, 24] support the general application of MR-directed ultrasound in newly diagnosed MR imaging BI-RADS 3 lesions. The value of MR-directed ultrasound is underscored by a recent meta-analysis that reported a pooled discovery rate of malignant findings detected by MR imaging as high as 79% (95% CI 71-87%) [36]. The same publication revealed a pooled detection rate for benign lesions of 52% (95% CI 44-60%). As a result, a substantial rate of benign MR imaging BI-RADS 3 lesions may be identified and followed up by ultrasound [36].

Of major interest are follow-up frequencies and intervals for MR imaging BI-RADS 3 lesions. The ACR BI-RADS recommends six-month intervals over a course of 24 months, an approach adapted from conventional imaging. While a reduced number of follow-up examinations are preferable in terms of cost-effectiveness and patient compliance, our results provide supporting evidence for the current BI-RADS instructions. This is because malignant lesions were diagnosed at all time points up to 24 months after the initial diagnosis, although no study provided dedicated data on lesion appearance during each six-month imaging follow-up, and, furthermore, most authors did not specify whether six-month intervals were performed in all patients or not. This results in a research gap where dedicated data might further corroborate or refute the necessity for six-month interval follow-up over 24 months.

In conclusion, our meta-analysis identified strongly heterogeneous prevalence of MRI BI-RADS 3 lesions while pooled malignancy rates in general met BI-RADS benchmarks (<2%). Malignancy rates varied according to lesion features, exceeding 2% in non-mass lesions. According to the lack of sufficient data, twenty-four-month surveillance is required to detect all malignant lesions.
